# Are Temperate Canopy Spiders Tree-Species Specific?

**DOI:** 10.1371/journal.pone.0086571

**Published:** 2014-02-20

**Authors:** Anne-Christine Mupepele, Tobias Müller, Marcus Dittrich, Andreas Floren

**Affiliations:** 1 Biometry and Environmental System Analysis, University of Freiburg, Freiburg, Germany; 2 Department of Bioinformatics, University of Würzburg, Würzburg, Germany; 3 Department of Animal Ecology and Tropical Biology, University of Würzburg, Würzburg, Germany; University of Western Ontario, Canada

## Abstract

Arboreal spiders in deciduous and coniferous trees were investigated on their distribution and diversity. Insecticidal knock-down was used to comprehensively sample spiders from 175 trees from 2001 to 2003 in the Białowieża forest and three remote forests in Poland. We identified 140 species from 9273 adult spiders. Spider communities were distinguished between deciduous and coniferous trees. The richest fauna was collected from *Quercus* where beta diversity was also highest. A tree-species-specific pattern was clearly observed for *Alnus*, *Carpinus*, *Picea* and *Pinus* trees and also for those tree species that were fogged in only four or three replicates, namely *Betula* and *Populus*. This hitherto unrecognised association was mainly due to the community composition of common species identified in a Dufrene-Legendre indicator species analysis. It was not caused by spatial or temporal autocorrelation. Explaining tree-species specificity for generalist predators like spiders is difficult and has to involve physical and ecological tree parameters like linkage with the abundance of prey species. However, neither did we find a consistent correlation of prey group abundances with spiders nor could differences in spider guild composition explain the observed pattern. Our results hint towards the importance of deterministic mechanisms structuring communities of generalist canopy spiders although the casual relationship is not yet understood.

## Introduction

Spiders are a prominent group of predators in ecosystems and have received increasing interest in research during the last years [1]–[4]. In temperate forests, canopy spiders contribute between 4% and 12% to the arboreal arthropod fauna playing an important role in the regulation of insect populations [5], [6]. Considering the canopy in ecological research is therefore expected to foster our understanding of the relationships and species interactions that govern ecosystem processes and ecosystem function. Understanding the assembly rules of spider communities can provide valuable information towards these processes and is therefore an important issue in basic ecological research.

Spiders are mostly generalist predators and canopy spiders are assumed to be little associated with their host trees, although habitat structure and microclimatic conditions influence the distribution of many spiders [7]–[9]. Previous work has emphasised that structural and abiotic conditions between deciduous and coniferous trees can result in distinguishable communities of spiders [10], [11]. Apart from that, spiders are not known to discriminate between tree species. Prey availability is another factor potentially influencing the distribution of spiders and community composition but the evidence is contradictory indicating positive as well as negative relationships [7], [11]–[13].

Our work aims at investigating the functional importance of canopy spiders, their diversity and the mechanisms structuring spider communities in trees. We used guild composition as a proxy for species function in communities. In order to sample arboreal spiders as comprehensive as possible we used insecticidal knock-down (fogging), which is currently the best method to get a quantitative view of spider abundance and guild structure in tree crowns [14]–[16]. Fogging makes it also possible to collect canopy arthropods in a tree-specific way by exactly positioning the collecting sheets beneath the study tree. This offers the possibility to investigate the structure of spider communities on individual trees. Our investigation was carried out in the Polish Białowieża forest and is based on 175 fogged heterospecific trees in different forests. This allowed us to perform a rigorous analysis of the distribution of canopy spiders. In particular we were interested in answering the following questions: 1) Do spider communities differ between tree species and how is this reflected on the beta diversity level? 2) How consistent is guild composition between tree species? 3) Is the abundance and composition of arboreal prey a predictor of the composition of spider communities?

## Methods

### Study area

Canopy spiders were collected in the Białowieża forest in Eastern Poland (52°30′–53°00′N; 23°30′–24°25′E) which is considered one of the last pristine lowland forests in Central Europe covering 1500 square kilometres [17]. The forest harbours 25 species of trees belonging floristically to the formation Tilio carpinetum [18]. With the exception of the strictly protected pristine areas, the forest is managed in near-to-nature manner. Furthermore, we performed foggings in three other forests, namely in Kampinoski (10 *Q. robur*), Borecka (4 *P. abies*, 3 *Q. robur*, 3 *C. betulus*) and Nurzec (7 *Q. robur*). All forests were at least 50 kilometres away from the Białowieża area (Figure S1 in File S1). Field work was carried out in the years 2001 (78 trees), 2002 (44 trees) and 2003 (53 trees) with the permission of J. Lugovoj, the head of the Hajnowka forest district.

A total of 175 trees were fogged of which 98 were oaks (*Q. robur* L., Fagaceae). *Q. robur* is a common tree species harbouring one of the most diverse arthropod faunas [19], [20]. This explains why oak trees were in the focus of this project. The fogged oak trees were of 30, 50, 80, 170 and larger 200 years. Other tree species were fogged in lower numbers in order to get an impression about spider diversity on heterospecific trees (see Table 1). These were 18 *Carpinus betulus* L. (Betulaceae, between 80 and 120 years); 10 *Alnus glutinosa* (L.) Gaertner (Betulaceae, of about 100 years); 4 *Betula pendula* Roth (Betulaceae, 60 years); and 3 *Populus tremula* L. (Salicaceae) of about 60 years. Furthermore, we collected spiders from 32 *P. abies* (L.) H. Karst. (Pinaceae, trees were 8, 30 and 100 years) and 10 *Pinus sylvestris* L. (Pinaceae, 100 years). For more information we refer to [5].

**Table 1:** Diversity of spider communities.

	Qr	Cb	Ag	Bp	Pt	Pa	Ps	All
**Foggings**	98	18	10	4	3	32	10	175
**Species**	118	45	42	31	24	51	29	140
**Abundance**	6616	822	224	85	63	1296	167	9273
**Most abundant species**	1038	271	37	10	8	198	58	1253
**Singletons**	34	14	21	13	7	12	13	40
**% Singletons**	29	31	50	42	29	24	45	29
**Shannon**	3.15	2.69	2.97	3.12	2.99	2.96	2.48	3.28
**Pielous Evenness**	0.66	0.71	0.79	0.91	0.94	0.75	0.74	0.66
**RAF (ind = 63)**	24	19	22	27	24	20	18	24
**RAF (ind = 167)**	36	27	36			29	29	37

Diversity of spider communities collected by insecticidal knock down from deciduous and coniferous trees in Poland. Rarefaction values (RAF) computed on standardized individual numbers (ind) allow direct comparison suggesting large differences in species diversity among tree species (Qr = *Q. robur*, Cb = *C. betulus*, Ag = *A. glutinosa*, Bp = *B. pendula*, Pt = *P. tremula*, Pa = *P. abies*, Ps = *P. sylvestris*).

### Sampling method

Arboreal arthropods were collected by means of insecticidal knock-down using natural pyrethrum as an insecticide. Fogging samples free-living, mobile arthropods in a comprehensive and tree-specific way. Tree specificity is achieved by placing the collecting sheets beneath the crown projection area of the study tree excluding branches from neighbouring trees. For technical details see [15]. All foggings were performed in June under similar climatic and phenological conditions allowing comparability of results between years.

### Species identification, guild composition and prey abundance

Only adult spiders were identified to species level and used in the analyses. Voucher specimens are kept in the collection of AF. Differences in the functional composition of spider communities were analysed via guild composition. Spiders were classified according to their foraging strategies following [21]. A more recent classification which uses a different division of guilds [2] did only marginally affect our data (only four of the 1029 hunting spiders were distinguished of other hunting spiders) so that we kept to the original approach. Web-building spiders were separated between space-web weavers (Theridiidae, Dictynidae), orb-web weavers (Araneidae, Tetragnathidae, Theridiosomatidae, Uloboridae) and tangle weavers (Linyphiidae). Among hunting spiders we distinguished ambushers (Philodromidae, Thomisidae), stalkers (Salticidae, Mimetidae) and foliage runners (Clubionidae, Anyphaenidae, Sparassidae). Ground runners (Gnaphosidae and Lycosidae) contributed less than 1% to all canopy spiders and were excluded from the guild analysis. We used the fogging data to correlate spider abundance with prey abundance. Diptera, Hymenoptera, Psocoptera, Homoptera, Heteroptera and Coleoptera are common spider prey [22], [23] and their abundance was used to test whether prey availability had an effect on spider community composition.

### Statistical analysis

Analyses were performed in R version 2.15.2 [24], using the packages vegan, alphahull and labdsv [25]–[27]. Spider communities were analysed by the Shannon diversity, Pielous evenness and rarefaction. Further we used correspondence analyses (CA), an unconstrained ordination method, to structure species-abundance data [28]. We calculated group centroids and inner alpha shapes (circles around related groups of data excluding extreme values; alpha set to 0.8) in order to better separate groups with overlapping data points. The factors height of tree, girth in breast height, forest age and distance between trees, were recorded in the field and tested by the function envit as implemented in the vegan package. This function correlates ordination scores against the factors [26]. We repeated the analysis without singletons and tourist species identified according to [29] and with presence-absence data to test for robustness of the results. Guild composition was plotted as box-plots with notches showing the 95% confidence interval of the median on a log transformed y-axis. We used **an**alysis **o**f **sim**ilarity (ANOSIM) for testing differences in guild composition between tree species. The function operates on a dissimilarity matrix based on the Morisita-Horn index. If two groups of sampling units are really different in their composition, then dissimilarities between groups ought to be greater than those within groups [26]. Due to multiple comparisons significance levels were corrected according to Benjamini-Höchberg. Differences in the abundance and frequency distribution of species were tested by a Dufrene-Legendre indicator species analysis [30]. Only species with at least 10 individuals were considered in this analysis. The arthropod numbers obtained by fogging were used as a surrogate of prey availability and tested for correlation with spider guild composition (Spearman rank correlation).

#### Beta diversity

Similarity between communities was analysed by calculating the Morisita-Horn (MH) beta diversity (1). This index is independent of sample size and widely used in ecology [31], [32]. It is included in the vegan package and computed as a dissimilarity index (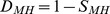).
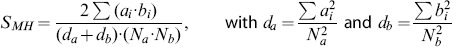
(1)Where 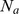 is the total number of individuals at site A, 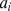 is the number of individuals of the ith species at site A, 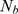 is the total number of individuals at site B and 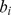 is the number of individuals of the ith species at site B. The index ranges between 0 and 1. We calculated beta diversity for each tree species to compare similarity of spider communities. In order to correct for uneven sampling calculations were performed on a sub-sample size of 10 randomly chosen conspecific trees, the smallest common number of trees (sampling without replacement). Due to low sample size, *B. pendula* (n = 4) and *P. tremula* (n = 3) were not included in this analysis. The whole procedure was repeated 1000 times. Differences in beta diversities were visualised in a density plot and tested by an ANOVA model with tree species as predictor and beta diversity as response variable.

We tested whether diversity of spider communities was influenced by spatial autocorrelation by applying a Mantel-Test based on Pearson correlation with 1000 permutations [33].

## Results

**Figure 1:**
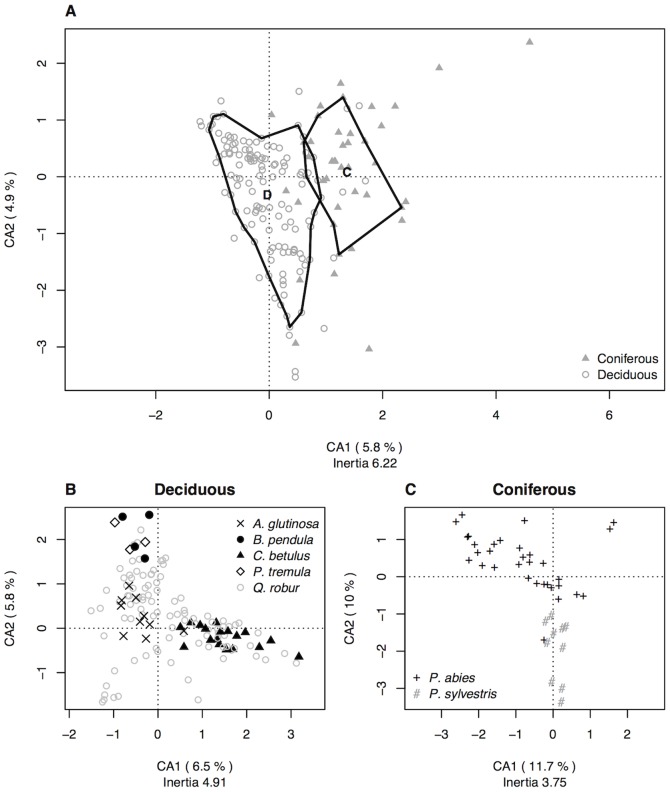
Correspondence analyses showing the distribution of spiders on the fogged trees. Spider communities on deciduous trees and conifers are clearly separated exhibiting a larger similarity within than between groups (A). For both deciduous (B) and coniferous (C) trees, tree-species-specific patterns were identified. No such pattern was found for the oak trees.

A total of 36036 spiders were collected among which were 26763 (74%) juveniles. The 9273 adults were sorted to 140 species and used in the analysis. All species represent 10.7% of the 1313 species known from Central Europe and 17.7% of the 792 species known from Poland [34]. Numbers of adult spiders varied largely between trees both in respect to individuals and species. On average we collected 52.9 (standard deviation(SD): 67.4) spider individuals and 13.3 (SD 7.3) spider species per tree. Table 1 shows that species numbers per tree species positively correlated with individual numbers which in turn correlated with the number of foggings. Correspondingly, most species were collected from the oaks (118 species or 84.3% of all 140 species), followed by spruces, hornbeam and alder trees. All trees were dominated by few abundant species and characterized by a large proportion of singletons which provided on average 35.6% (SD 9.8). Spider diversity was high on *Betula* and *Populus* due to a high evenness but low on hornbeam and pine (Table 1). Deciduous trees harboured significantly more species per tree (mean = 14.3) than coniferous trees (mean = 10.1; Mann Whitney U-test: W = 3762.5, *P*<0.001) and both groups were clearly discriminated in a correspondence analysis (Figure 1A). This pattern was robust and did not change after excluding singletons and tourist species from the analysis (Figure S3B in File S1). On account of these clear differences the subsequent analyses were carried out separately between deciduous and coniferous trees.

We tested the importance of the factors in the ordination. Spider communities on conspecific trees were highly correlated (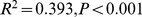) as were communities collected from deciduous and coniferous trees (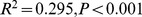). In addition, tree age (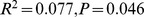) and GBH (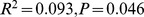) was weakly significant.

### Spider communities on deciduous and coniferous trees

From the 133 deciduous trees a total of 7810 spiders were collected and sorted to 132 species representing 84.2% of all spider individuals and 94.3% of all species. In a CA the oak spider communities showed no clustering in respect to year of sampling or any other recorded factor. In contrast, the spider communities of the other deciduous trees were grouped together within the oak-point cloud indicating a tree-species-specific association (Figure 1B, 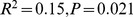). On the other hand, the oak trees harboured most of all spider species collected in the field (Table S2 in File S1) and showed no tree-species-specific pattern of community organisation. This pattern became clearer in the ordinations when the oaks are excluded from the analysis (Figure S2 in File S1). Communities on *C. betulus* differed largest from the other tree species and were separated on the first axis of the CA while communities on birch, poplar and alder trees were separated on axis 2. This result was not due to spatial autocorrelation (Mantel test r = 0.06, *P* = 0.081 (including oaks); r = −0.11, *P* = 0.87 (excluding oaks)). The robustness of the tree-species-specific clustering is additionally supported by the CA on presence-absence data (Figure S3C in File S1) as well as after excluding 49 singletons and 17 tourist species, which resulted in an increase of the explanatory power of the first two axes from 6.4% to 18.9% (Figure S3D in File S1).

From the 42 coniferous trees 1463 spiders, 15.8% of the total, were sampled and sorted to 63 species representing 45% of the total. Spider communities on spruce and pine were distinguished in a CA on axis two while axis one represented the age gradient of the spruce trees (Figure 1C, tree species: 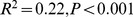; age: 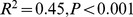). All fogged pine trees were of similar age. One extremal spruce tree was placed in the pine tree cluster. The data were weakly autocorrelated (Mantel-test, r = 0.15, *P* = 0.052). Again this pattern was supported by a CA on presence-absence data which reflected the tree-species-specific effect on axis one. This pattern became more pronounced after the exclusion of 20 singletons and two tourist species (Figure S3E,F in File S1).

**Figure 2:**
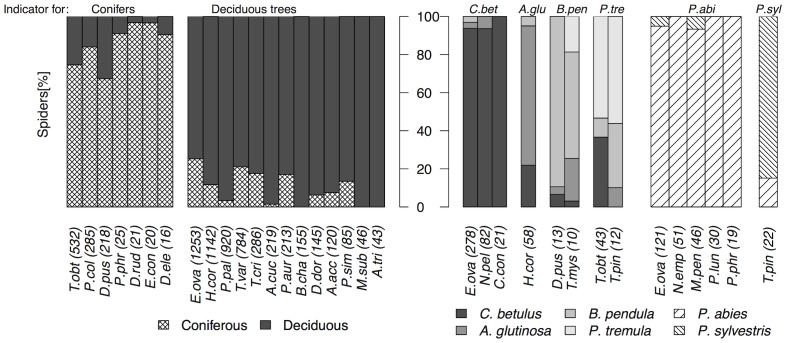
Proportion of indicator species. Species distinguishing deciduous from coniferous trees (left side) and species identified for individual tree species comparing deciduous trees that showed a tree-species-specific pattern (*C. betulus*, *A. glutinosa*, *B. pendula*, *P. tremula*) as well as coniferous trees (right side of the y-axis). Number of spider specimens are in brackets. Abbreviations as in Table S2 in File S1.

In the following we tested which species differed in abundance and frequency distribution between deciduous and coniferous trees as well as between individual tree species. Analysis indicated that 20 species were associated with either deciduous or coniferous trees, among them the most common species in our investigation *Enoplognatha ovata*, *Hypomma cornutum* and *Paidiscura pallens* on deciduous and *Tetragnatha obtusa* on coniferous trees (Figure 2, Table S1 and S2 in File S1). We identified eight species on the deciduous trees, *C. betulus*, *A. glutinosa*, *B. pendula* and *P. tremula*, as indicator species (Figure 2, Table S1 in File S1). For example, *E. ovata* were found with 271 adult individuals on *C. betulus* while *H. cornutum* was found in highest numbers on *A. glutinosa*. *P. abies* and *P. sylvestris* were distinguished by six species. Tree-species-specific associations of spiders were caused by differences in the abundance distribution of the common species. *E. ovata* and *Theridion pinastri* were identified as indicator species for both *C. betulus* and *P. abies*. No indicator species were identified for the oak trees.

**Figure 3:**
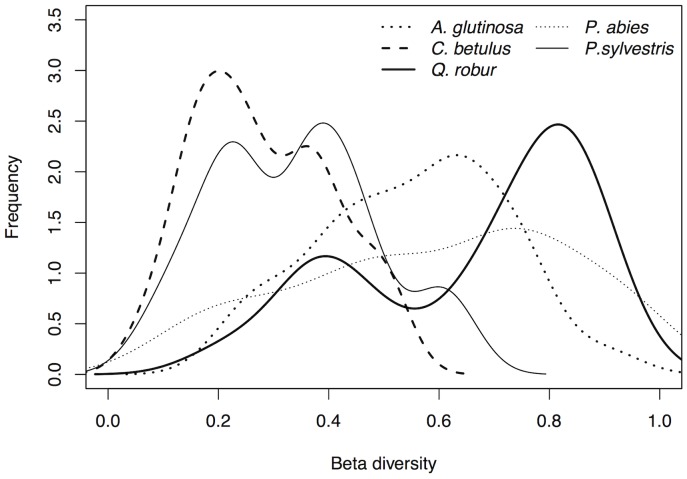
Beta diversity of spider communities. Comparison of spider communities per tree species on the beta diversity level. The density distribution of Morisita-Horn values of 1000 permutations of ten randomly chosen trees visualizes differences in community composition independent of sample size. Beta diversity was largest on oak, followed by spruce and alder trees.

Comparing beta diversities of spider communities between tree species requires controlling for differences in sample size. In order to achieve this we calculated the mean Morisita-Horn diversity on a subsample of ten randomly chosen *Q. robur*, *C. betulus*, *A. glutinosa*, *P. abies* and *P. sylvestris* trees and repeated this procedure 1000 times. Subsequent ANOVAs confirmed significant differences in species composition between tree species (ANOVA, F = 40.35–116.12; df = 4; *P*<0.001). Figure 3 shows highest beta diversity for the spider communities collected from the oaks (the Morisita-Horn (MH) index peaked at 0.82) which differed significantly from the other tree species (Tukey post-hoc: *P*<0.001) except for *P. abies* (MH = 0.74) where index values were found to significantly differ only in 23% of all permutations. Mean beta diversity for spiders of the alder trees was 0.63, for pines 0.39 and for hornbeam 0.2. We also tested whether beta diversity on the oaks was correlated with geographic position but found no significant relationship (Mantel test, r = 0.064, *P* = 0.088).

### Guild composition

**Figure 4:**
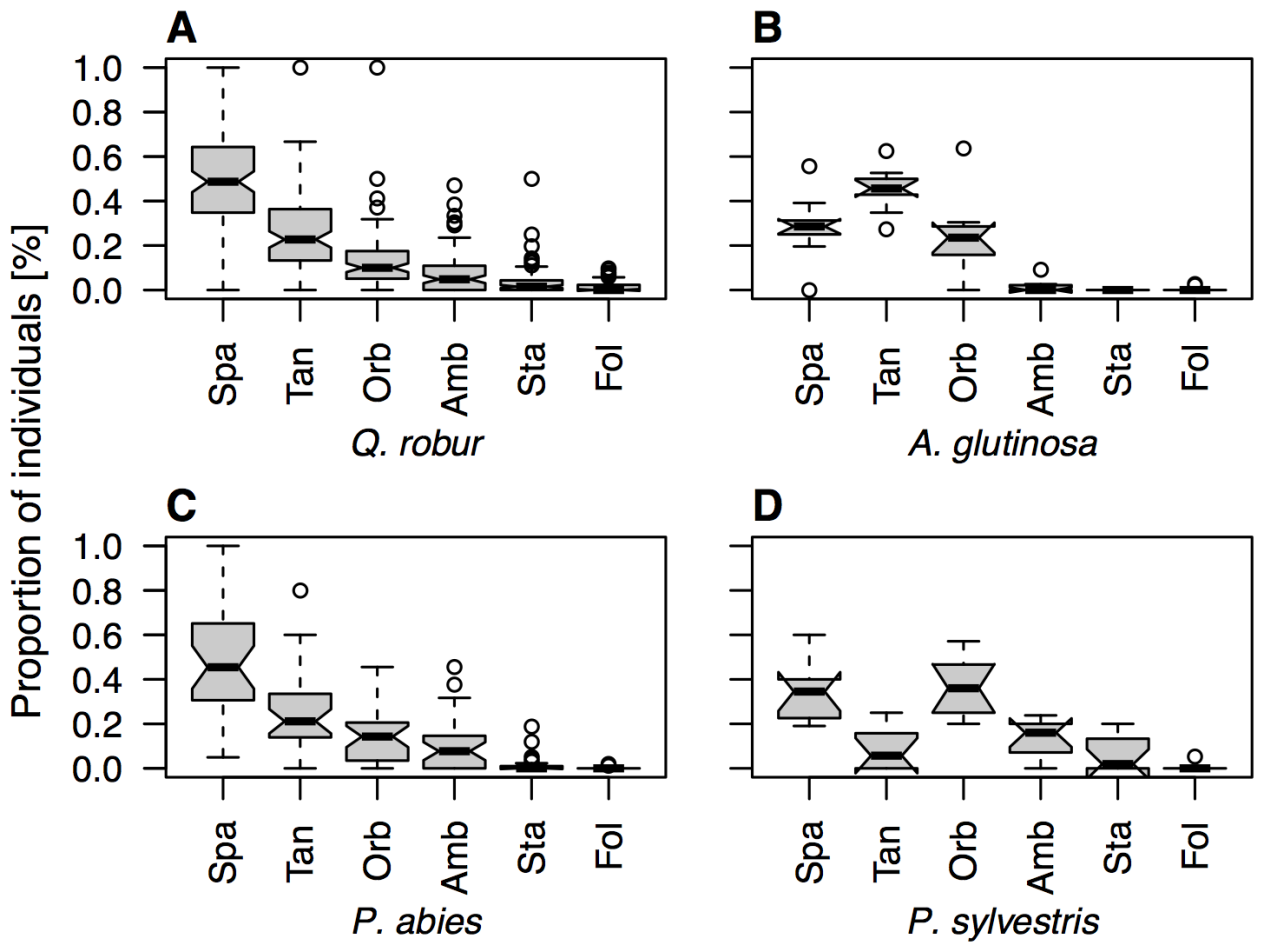
Guild distribution. Box-plots showing characteristic types of distributions of guilds on different tree species. Abbreviation: Space-web weavers = Spa, tangle weavers = Tan, orb-web weavers = Orb, ambushers = Amb, stalkers = Sta, foliage runners = Fol. Guild composition was uniform on most trees (*Quercus*, *Carpinus*, *Betula* and *Picea*), while *Alnus* and *Pinus* were dominated by tangle and orb-web weavers.

All trees were dominated by web-building spiders which comprised 89.8% of all specimens. Hunting spiders accounted for 10.2% of the total (Figure 4). Among web-building spiders, space-web weavers were most abundant providing between 29% and 51% of all spiders per tree species. Highest numbers of space-web weavers were collected from *C. betulus* and *Q. robur*, which was mainly due to the Theridiidae *E. ovata* (Figure 2, Table S2 in File S1). Space-web weavers were outnumbered by tangle weavers only on the alder trees which was dominated by *H. cornutum*, Linyphiidae. Orb-web weaving spiders were found dominant on poplar and pine trees. Ambushers were the most frequent of all hunting spiders reaching highest proportions on the pines. Generally, stalkers and foliage runners contributed only few specimens to the communities. Guild arrangement was similar on *Quercus*, *Carpinus*, *Betula* and *Picea*. *Alnus* trees differed by high proportions of tangle weavers while *P. sylvestris* was characterized by orb-web weavers. ANOSIM confirmed significant differences between tree species, see Figure 4. *P. sylvestris* differed significantly from other tree species (*P. sylvestris* versus *A. glutinosa* (R = 0.7, *P*<0.001); *P. sylvestris* versus *Q. robur* (R = 0.32, *P* = 0.004); *P. sylvestris* versus *P. abies* (R = −0.13, *P* = 0.047), in particular see Figure S4 in File S1).

### Is tree-species specificity caused by prey availability?

We tested whether guild composition within tree-species-specific communities was correlated with the abundance of particular groups of prey taxa but again we found no consistent pattern. From all possible 252 correlations 32 were significant after Bonferroni correction. Diptera were positively associated with space-web builders (Spearman rank correlation 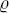 = 34.17, *P* = 0.0034) and tangle weavers (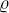 = 32.95, *P* = 0.019) on *Alnus* and also with ambushers on *Carpinus* (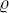 = 29.47, *P* = 0.0012) and space-web builders on *Picea* (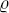 = 21.03, *P* = 0.016). Furthermore, Coleoptera were positively correlated with space-web builders (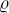 = 20.05 *P* = 0.034), orb-web weavers (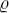 = 27.49, *P*<0.001), tangle weavers (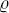 = 20.37, *P* = 0.027) and ambushers (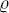 = 26.46, *P*<0.001) on *Picea* while Heteroptera correlated with space-web builders on *Picea* (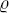 = 24.04, *P* = 0.001). On *Quercus*, prey abundance was positively correlated with most guilds with the exception of Psocoptera, which showed no correlations at all.

## Discussion

Canopy studies in temperate regions are still underrepresented in ecological research although trees harbour a large proportion of forest species diversity which greatly influences ecological processes like decomposition, predation or herbivory. Among arthropods spiders are an abundant group of predators and regularly found in high abundance in the trees [16], [20], [35]. However, quantitative data are difficult to get. Insecticidal knock down has improved this situation greatly promising to deliver data on one of the less well-documented groups of arthropods [36]. It does not only enable access to a habitat which is difficult to reach but allows to picture spider communities including their functional composition [5]. By fogging a large number of trees we collected not only remarkable spiders from the canopy and could provide a lot of new information on spider distribution [37], [38] but we detected that spiders can be distributed in a way suggesting a tree-species-specific association.

### Tree-species specificity of spider communities

In correspondence with the little knowledge available on spider distribution in forest canopies we here provide substantial evidence based on the largest data set available today that arboreal spider communities in temperate forests differ significantly between conifers and deciduous trees [11], [39]. Experimental field work suggests that this is a consequence of the microclimatic and structural differences between conifers and deciduous trees caused for example by leaf shape, branch and leaf density or bark structure [8], [10]. Furthermore, we were also able to identify the spider species that were indicative for this separation. These were abundant and common species which are usually collected in large numbers like *E. ovata*, *H. cornutum*, *P. pallens* or *Theridion varians* (Figure 2, Table S2 in File S1) on deciduous trees. In contrast, only few species showed an association with conifers, like *Dendryphantes rudis* or *Entelecara congenera* or the common *Philodromus collinus* or *Philodromus praedatus* of which the latter two species were not yet known to preferentially occur on conifers.

*Quercus* was the focal tree of our study and fogged in largest numbers. The oaks harboured an especially rich fauna of spiders comprising almost 85% of all spider species collected, including most of the conifer preferring species. High diversity was also the reason why no indicator species and no tree-species-specific community could be identified (Figure 1A, Table S1 in File S1). Although the spider diversity is positively correlated with sampling effort, community composition on *Quercus* was found most variable and showed highest beta diversity distinguishing the oak communities from all other trees (Figure 3). Other taxa of arthropods are also remarkably rich on the oaks; a pattern that is not yet understood [19], [40], [41]. Spider richness was coupled with high beta diversity and the space defined by the two main axes of the correspondence analysis was completely filled with oak trees masking the tree-species-specific association on the other trees (Figure 1B).

A certain degree of tree-species specificity in spider distribution has already been observed earlier [42]. Larrivee and Buddle had investigated spiders on sugar maple and American beech in hardwood forests of Canada [43], [44]. Overall, these are individual findings based on a few hand beating samples which did also reach only a few meters into the canopy, but which did not cover the whole tree crowns. It is for this reason why tree-species specificity had not been noted and discussed thoroughly. Arguably difficult accessibility into the canopy has also contributed to this situation and still field work is often restricted to the lower stratum of trees. Thus, for the first time fogging provides a methodological approach to assess and analyse community composition using quantitative data.

We show that many spider species can distinctly differ in their distribution between tree species forming stable communities in space and time. It is important to notice that spider communities showed only weak temporal and spatial auto-correlation also demonstrating that tree-species specificity was not a methodical artefact. This applies even to those tree species that were fogged in low numbers suggesting that the association of spiders with their host trees can be much more pronounced than between deciduous and coniferous trees [8]. Obviously, tree-species specificity was not the outcome of a chance process because all trees fogged clustered independently together for all three years and for all forest types. This pattern was robust and did not change after excluding singletons and tourist species emphasizing how important the common species were in characterizing communities. This is furthermore supported by the indicator species analysis (Figure 2, Table S2 in File S1). Remarkably, *E. ovata* and *T. pinastri* were identified as indicator species for both a broad-leaf and a conifer tree species. This suggests that species are not directly associated with their ‘host trees’ but that their distribution follows small-scale habitat specific differences generated for example by climatic or structural differences e.g. tree height, leaf size, bark structure or branching patterns. As many of these factors correlate with the age of forest stands we found ‘age’ of significant importance for community composition, but without affecting the general pattern of spider distribution.

### Spider prey distribution and guild composition in the canopy

Our data allowed us to analyse the factor prey distribution in greater detail which is often hypothesized to influence spider communities and guild composition [8], [39]. However, this factor did not result in a uniform picture helping and explaining spider distribution although prey availability was assessed with large accuracy by using the fogging data. Besides prey availability tree-species specificity of associations might be explained by indirect association between predators and their prey. However, spiders are feeding generalists and only few prey specializations are known mainly among hunting spiders [7], [22], [45]. Respective associations should also become obvious in the guild composition. However, there is no meaningful evidence towards such relationships. In contrast, guild composition was similar between oak and spruce trees (Figure 4), while alder and especially pine trees showed deviating patterns. Diptera, for example, are among the most abundant arthropods in the canopy [20] and are an important group of prey [22], but varied inconsistently between trees and guilds. Moreover, significant correlations were found for Heteroptera and Coleoptera for which no specialisation has yet been recorded. In contrast, preferred food organisms like little chitinised arthropods as Psocoptera, Aphids or parasitic Hymenoptera [22] showed no correlation at all. Altogether, our data do not support the hypothesis that prey abundance is a driver of tree-species specificity of spider communities.

Investigations indicate that neglecting juveniles, which can provide more than 70% of all individuals [46] might distort the results. Juveniles are often excluded because they are difficult to identify. More specific studies particularly on the importance of tree structural properties are required to allow a more fine graded ecological classification of species in order to clarify the assembly rules of communities and to better characterize the ecological role of canopy spiders in forest ecosystems.

## Conclusion

The surprising observation that generalist predators discriminate between tree species suggests that the relationship of spiders with their host trees is more complex than hitherto assumed. Here we show how strong and stable such a pattern can be in space and time. There is little evidence that community composition and guild arrangement is influenced by availability of prey organisms. In contrast, our data hint towards the highly complex interrelationship between local habitatspecific and regional e.g. climate specific conditions which need to be disentangled in greater detail.

## Supporting Information

File S1 **Supporting figures and tables.** **Figure S1.** Map displaying distance between study trees in Poland. The Białowieża forest was the main research area and all forest plots were embedded in the forest matrix. We also collected spiders in the forests of Kampinoski, Borecka and Nurzec which were at least 50 km away from the Białowieża forest. **Figure S2.** Correspondence analysis displaying deciduous trees with a tree-species-specific pattern. **Figure S3.** Correspondence analyses displaying presence-absence data (a,c,e) and data without singeltons and tourists (b,d,f) for all data (a,b) and deciduous (c,d) and coniferous trees (e,f) separately. **Table S1.** Dufrene-Legendre indicator species analysis identifying characteristic species for deciduous and coniferous trees and for tree species. **Figure S4.** Guild composition differed between tree species as demonstrated by analysis of similarity (ANOSIM). Box-plots show dissimilarity of spider community composition between and within trees. After correcting for multiple testing according to Benjamini-Höchberg, significant differences in guild composition were found for the comparisons of *P. sylvestris* with *A. glutinosa*, *Q. robur* and *P. abies* (grey). **Table S2.** Abundance of all 140 spider species sorted according to their total abundance. Abbrev = Abbreviation of species names. A.g. = *A. glutinosa*, B.p. = *B. pendula*, C.b. = *C. betulus*, P.t. = *P. tremula*, Q.r. = *Q. robur*, P.a. = *P. abies*, P.s. = *P. sylvestris*.
(PDF)
