# Supplementary material for: Are Temperate Canopy Spiders Tree-Species Specific?
Source: PLoS One. 2014 Feb 20;9(2):e86571. doi: 10.1371/journal.pone.0086571 (PMC3930551; doi:10.1371/journal.pone.0086571)
Supplement: File S1 — Supporting figures and tables. Figure S1. Map displaying distance between study trees in Poland. The Białowieża forest was the main research area and all forest plots were embedded in the forest matrix. We also collected spiders in the forests of Kampinoski, Borecka and Nurzec which were at least 50 km away from the Białowieża forest. Figure S2. Correspondence analysis displaying deciduous trees with a tree-species-specific pattern. Figure S3. Correspondence analyses displaying presence-absence data (a,c,e) and data without singeltons and tourists (b,d,f) for all data (a,b) and deciduous (c,d) and coniferous trees (e,f) separately. Table S1. Dufrene-Legendre indicator species analysis identifying characteristic species for deciduous and coniferous trees and for tree species. Figure S4. Guild composition differed between tree species as demonstrated by analysis of similarity (ANOSIM). Box-plots show dissimilarity of spider community composition between and within trees. After correcting for multiple testing according to Benjamini-Höchberg, significant differences in guild composition were found for the comparisons of P. sylvestris with A. glutinosa, Q. robur and P. abies (grey). Table S2. Abundance of all 140 spider species sorted according to their total abundance. Abbrev = Abbreviation of species names. A.g. = A. glutinosa, B.p. = B. pendula, C.b. = C. betulus, P.t. = P. tremula, Q.r. = Q. robur, P.a. = P. abies, P.s. = P. sylvestris. (PDF) [file pone.0086571.s001.pdf]

Supplement File S1

Are temperate canopy spiders tree-species specific?

Anne-Christine Mupepele, Tobias Müller, Marcus Dittrich, Andreas Floren

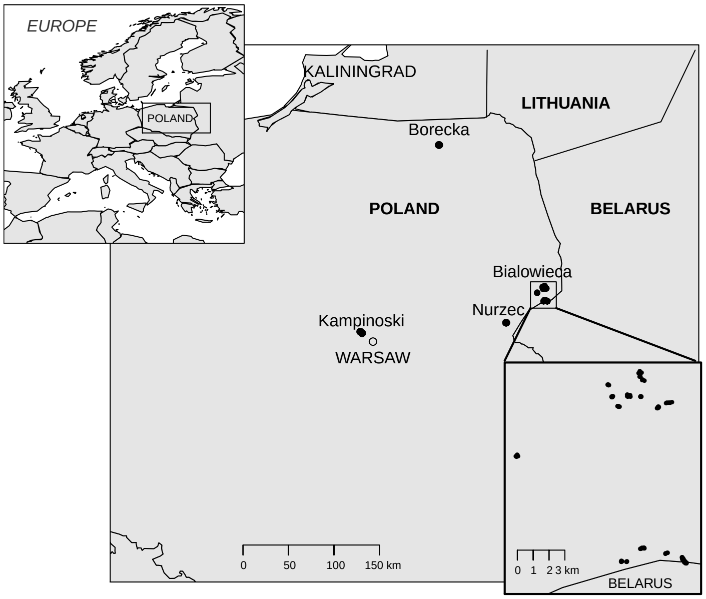

Figure S1 Map displaying distance between study trees in Poland. The Białowieża forest was the main research area and all forest plots were embedded in the forest matrix. We also collected spiders in the forests of Kampinoski, Borecka and Nurzec which were at least 50 km away from the Białowieża forest

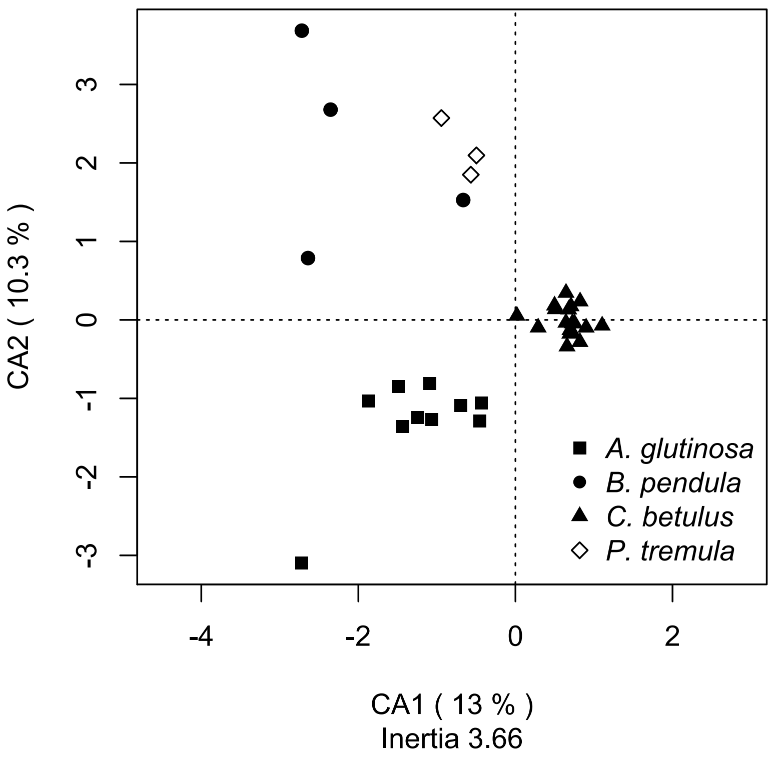

Figure S2 Correspondence analysis displaying deciduous trees with a tree-species-specific pattern

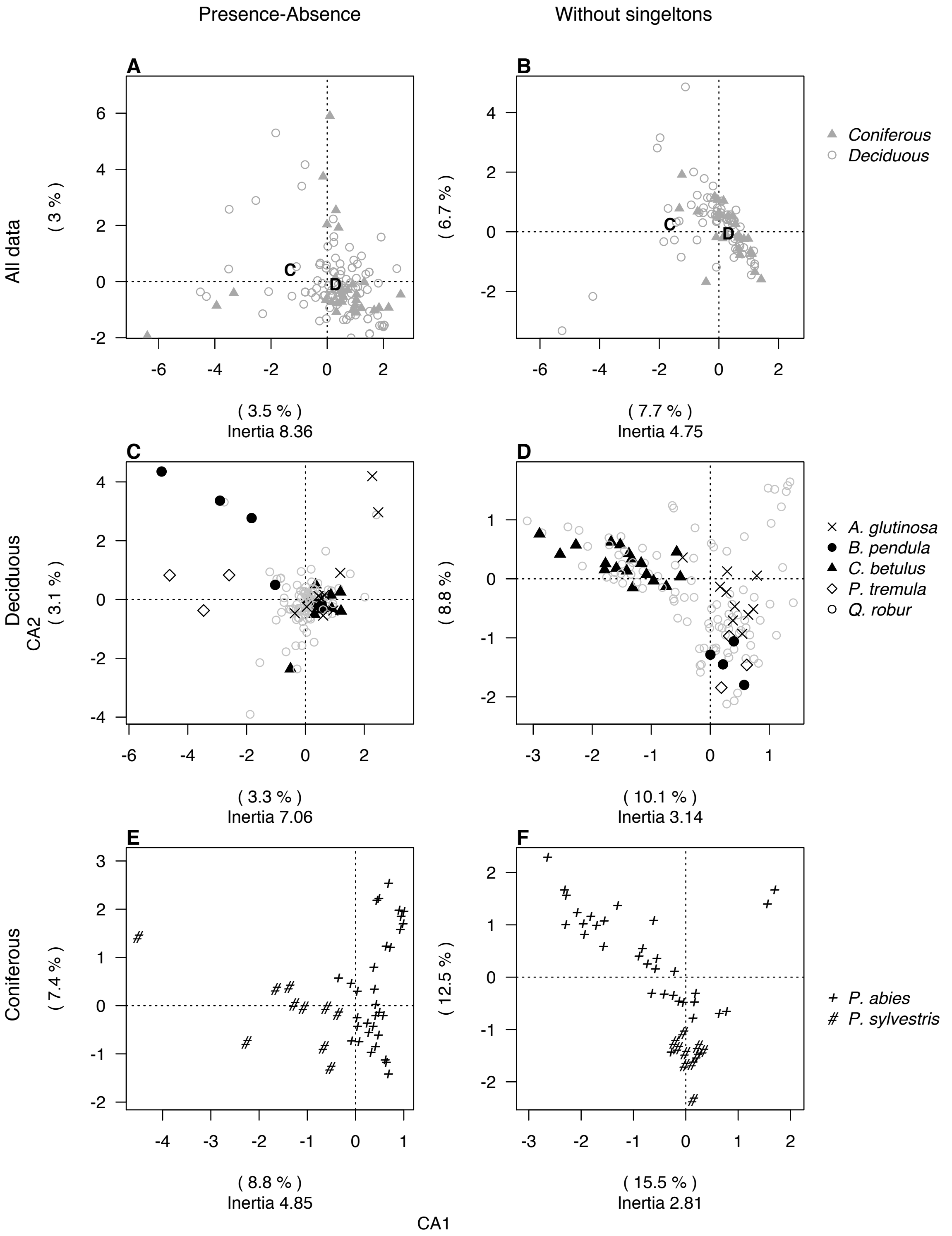

Figure S3 Correspondence analyses displaying presence-absence data (a,c,e) and data without singeltons and tourists (b,d,f) for all data (a,b) and deciduous (c,d) and coniferous trees (e,f) separately

Table S1 Dufrene-Legendre indicator species analysis identifying characteristic species for deciduous and coniferous trees and for tree species

**Spider species**		**Indicator value**	**p-value**
**Comparing deciduous and coniferous**			
Tetragnatha obtusa	Coniferous	0.533	< 0.001
Philodromus collinus	Coniferous	0.440	0.001
Dictyna pusilla	Coniferous	0.449	< 0.001
Pityohyphantes phrygianus	Coniferous	0.260	< 0.001
Dendryphantes rudis	Coniferous	0.230	< 0.001
Entelecara congenera	Coniferous	0.207	< 0.001
Dismodicus elevatus	Coniferous	0.108	0.009
Enoplognatha ovata	Deciduous	0.511	0.012
Hypomma cornutum	Deciduous	0.465	0.001
Paidiscura pallens	Deciduous	0.516	< 0.001
Theridion varians	Deciduous	0.647	< 0.001
Trematocephalus cristatus	Deciduous	0.521	< 0.001
Araniella cucurbitina	Deciduous	0.511	< 0.001
Philodromus aureolus	Deciduous	0.412	0.003
Ballus chalybeius	Deciduous	0.489	< 0.001
Diaea dorsata	Deciduous	0.296	0.006
Anyphaena accentuata	Deciduous	0.250	0.015
Parasteatoda simulans	Deciduous	0.228	0.021
Micaria subopaca	Deciduous	0.188	0.011
Araneus triguttatus	Deciduous	0.150	0.019
**Comparing C.b., A.g., B.p. and P.t.**			
Enoplognatha ovata	*C.betulus*	0.938	< 0.001
Neriene peltata	*C.betulus*	0.624	0.015
Cyclosa conica	*C.betulus*	0.667	0.007
Hypomma cornutum	*A.glutinosa*	0.731	0.001
Dictyna pusilla	*B.pendula*	0.894	< 0.001
Theridion mystaceum	*B.pendula*	0.559	0.012
Tetragnatha obtusa	*P.tremula*	0.533	0.030
Theridion pinastri	*P.tremula*	0.562	0.010
**Comparing P.a. and P.s.**			
Enoplognatha ovata	*P.abies*	0.682	0.005
Neriene emphana	*P.abies*	0.406	0.043
Moebelia penicillata	*P.abies*	0.554	0.014
Parasteatoda lunata	*P.abies*	0.469	0.020
Pityohyphantes phrygianus	*P.abies*	0.375	0.050
Theridion pinastri	*P.sylvestris*	0.679	< 0.001

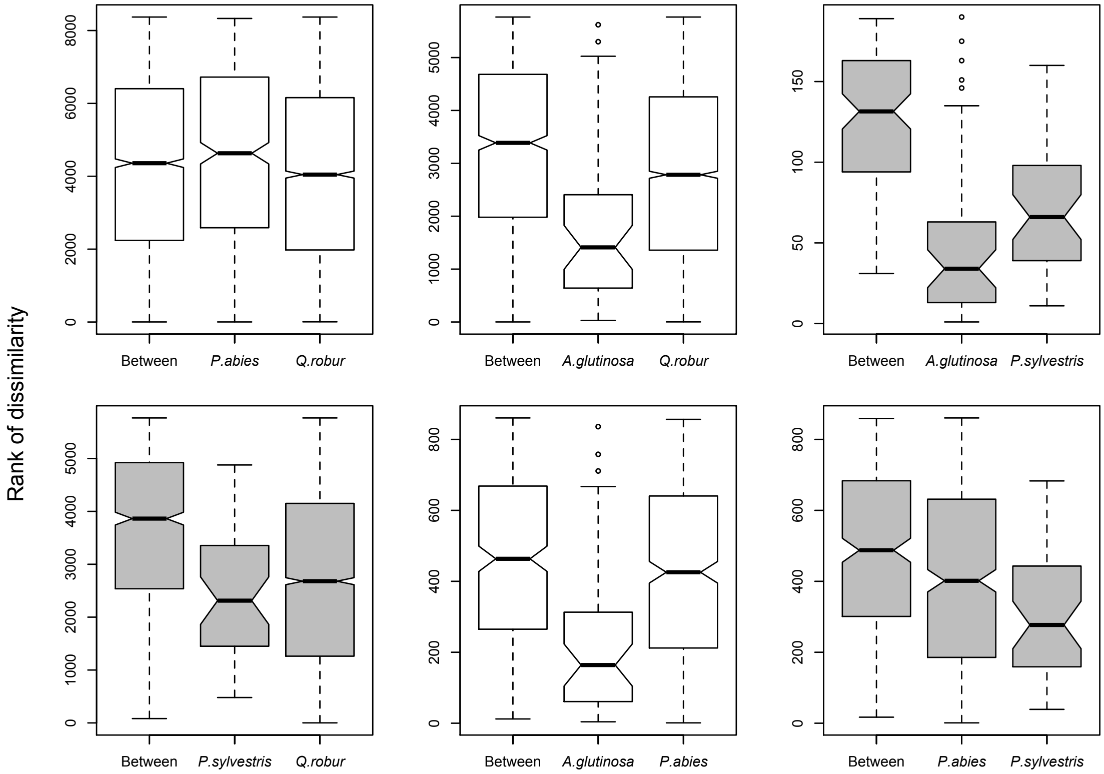

Figure S4 Guild composition differed between tree species as demonstrated by analysis of similarity (ANOSIM). Box-plots show dissimilarity of spider community composition between and within trees. After correcting for multiple testing according to Benjamini-Höchberg, significant differences in guild composition were found for the comparisons of *P. sylvestris* with *A. glutinosa*, *Q. robur* and *P. abies* (grey)

Table S2 Abundance of all 140 spider species sorted according to their total abundance. Abbrev=Abbreviation of species names. A.g. = *A. glutinosa*, B.p. = *B. pendula*, C.b.= *C. betulus*, P.t. = *P. tremula*, Q.r.= *Q. robur*, P.a. = *P. abies*, P.s.= *P. sylvestris*

Family	Spider species	Author	Abbrev	A.g.	B.p.	C.b.	P.t.	Q.r.	P.a.	P.s.
Theridiidae	*Enoplognatha ovata*	(Clerck, 1757)	E. ova	5	2	271	0	854	119	2
Linyphiidae	*Hypomma cornutum*	(Blackwall, 1833)	H. cor	37	1	20	0	1038	44	2
Theridiidae	*Paidiscura pallens*	(Blackwall, 1834)	P. pal	3	3	31	2	871	9	1
Theridiidae	*Theridion varians*	Hahn, 1833	T. var	18	2	55	5	644	58	3
Tetragnathidae	*Tetragnatha obtusa*	C.L. Koch, 1837	T. obt	0	2	33	8	233	198	58
Theridiidae	*Platnickina tincta*	(Walckenaer, 1802)	K. tin	12	5	29	3	279	143	15
Linyphiidae	*Trematocephalus cristatus*	(Wider, 1834)	T. cri	10	10	41	5	202	11	7
Philodromidae	*Philodromus collinus*	C.L. Koch, 1835	P. col	1	2	18	3	83	163	15
Linyphiidae	*Neriene peltata*	(Wider, 1834)	N. pel	3	0	79	0	107	62	0
Tetragnathidae	*Tetragnatha montana*	Simon, 1874	T. mon	25	0	43	0	151	29	0
Araneidae	*Araniella cucurbitina*	(Clerck, 1757)	A. cuc	9	1	16	1	191	1	0
Dictynidae	*Dictyna pusilla*	Thorell, 1856	D. pus	1	9	3	0	119	74	12
Philodromidae	*Philodromus aureolus*	(Clerck, 1757)	P. aur	0	4	7	2	187	7	6
Linyphiidae	*Moebelia penicillata*	(Westring, 1851)	M. pen	32	9	5	0	97	45	1
Theridiidae	*Theridion pinastri*	L. Koch, 1872	T. pin	3	4	0	5	139	8	14
Salticidae	*Ballus chalybeius*	(Walckenaer, 1802)	B. cha	1	1	22	0	132	0	0
Linyphiidae	*Neriene emphana*	(Walckenaer, 1842)	N. emp	3	0	21	0	78	51	0
Theridiidae	*Parasteatoda lunata*	(Clerck, 1757)	P. lun	5	0	25	0	86	30	0
Thomisidae	*Diaea dorsata*	(Fabricius, 1777)	D. dor	1	4	18	4	115	3	0
Anyphaenidae	*Anyphaena accentuata*	(Walckenaer, 1802)	A. acc	1	0	15	1	100	3	0

Table S2 Abundance of all 140 spider species sorted according to their total abundance. Abbrev=Abbreviation of species names. A.g. = *A. glutinosa*, B.p. = *B. pendula*, C.b.= *C. betulus*, P.t. = *P. tremula*, Q.r.= *Q. robur*, P.a. = *P. abies*, P.s.= *P. sylvestris*

Family	Spider species	Author	Abbrev	A.g.	B.p.	C.b.	P.t.	Q.r.	P.a.	P.s.
Araneidae	*Cyclosa conica*	(Pallas, 1772)	C. con	0	0	21	0	44	27	0
Theridiidae	*Parasteatoda simulans*	(Thorell, 1875)	P. sim	11	0	1	2	67	4	0
Theridiidae	*Theridion mystaceum*	L. Koch, 1870	T. mys	4	4	1	1	43	17	2
Philodromidae	*Philodromus praedatus*	O. P.-Cambridge, 1871	P. pra	0	0	3	0	4	61	0
Araneidae	*Araneus sturmi*	(Hahn, 1831)	A. stu	0	3	3	2	46	4	5
Araneidae	*Araniella alpica*	(L. Koch, 1869)	A. alp	0	2	4	0	51	4	0
Philodromidae	*Philodromus albidus*	Kulczynski, 1911	P. alb	0	0	2	0	56	0	0
Linyphiidae	*Neriene montana*	(Clerck, 1757)	N. mon	3	0	6	0	41	4	0
Gnaphosidae	*Micaria subopaca*	Westring, 1861	M. sub	2	3	0	0	42	0	0
Linyphiidae	*Diplocephalus picinus*	(Blackwall, 1841)	D. pic	1	0	1	0	41	3	0
Thomisidae	*Xysticus lanio*	C.L. Koch, 1835	X. lan	1	1	4	1	34	0	4
Araneidae	*Araneus triguttatus*	(Fabricius, 1793)	A. tri	0	0	3	4	36	0	0
Linyphiidae	*Meioneta innotabilis*	(O. P.-Cambridge, 1863)	M. inn	4	0	2	0	13	14	0
Linyphiidae	*Maso sundevalli*	(Westring, 1851)	M. sun	0	2	0	2	21	1	5
Araneidae	*Araniella opisthographa*	(Kulczynski, 1905)	A. opi	0	1	1	2	23	2	0
Linyphiidae	*Pityohyphantes phrygianus*	(C.L. Koch, 1836)	P. phr	0	0	0	0	6	19	0
Clubionidae	*Clubiona pallidula*	(Clerck, 1757)	C. pal	1	0	1	0	20	1	0
Linyphiidae	*Erigone atra*	Blackwall, 1833	E. atr	0	0	1	0	21	0	0
Salticidae	*Dendryphantes rudis*	(Sundevall, 1833)	D. rud	0	0	0	0	2	18	1
Tetragnathidae	*Metellina mengei*	(Blackwall, 1870)	M. men	0	0	3	0	13	5	0

Table S2 Abundance of all 140 spider species sorted according to their total abundance. Abbrev=Abbreviation of species names. A.g. = *A. glutinosa*, B.p. = *B. pendula*, C.b.= *C. betulus*, P.t. = *P. tremula*, Q.r.= *Q. robur*, P.a. = *P. abies*, P.s.= *P. sylvestris*

Family	Spider species	Author	Abbrev	A.g.	B.p.	C.b.	P.t.	Q.r.	P.a.	P.s.
Araneidae	*Stroemiellus stroemi*	(Thorell, 1870)	S. str	10	0	0	0	10	0	0
Linyphiidae	*Entelecara congenera*	(O. P.-Cambridge, 1879)	E. con	1	0	0	0	1	18	0
Clubionidae	*Clubiona marmorata*	L. Koch, 1866	C. mar	1	0	0	0	17	0	0
Linyphiidae	*Tenuiphantes tenebricola*	(Wider, 1834)	T. ten	0	0	3	0	14	1	0
Linyphiidae	*Dismodicus elevatus*	(C.L. Koch, 1838)	D. ele	0	1	0	2	1	11	1
Linyphiidae	*Porrhomma pygmaeum*	(Blackwall, 1834)	P. pyg	0	0	0	0	15	0	0
Theridiidae	*Anelosimus vittatus*	(C.L. Koch, 1836)	A. vit	0	0	1	0	13	0	0
Salticidae	*Pseudeuophrys erratica*	(Walckenaer, 1826)	P. err	0	0	1	0	11	0	1
Salticidae	*Salticus zebraneus*	(C.L. Koch, 1837)	S. zeb	1	0	0	0	11	1	0
Linyphiidae	*Gongylidium rufipes*	(Linnaeus, 1758)	G. ruf	0	0	1	0	12	0	0
Linyphiidae	*Erigone dentipalpis*	(Wider, 1834)	E. den	0	0	1	0	11	0	0
Theridiidae	*Dipoena torva*	(Thorell, 1875)	D. tor	2	0	0	0	8	0	0
Araneidae	*Gibbaranea gibbosa*	(Walckenaer, 1802)	G. gib	0	0	0	0	10	0	0
Linyphiidae	*Meioneta rurestris*	(C.L. Koch, 1836)	M. rur	0	0	0	0	7	2	0
Araneidae	*Larinioides patagiatus*	(Clerck, 1757)	L. pat	0	0	0	0	9	0	0
Salticidae	*Heliophanus dubius*	C.L. Koch, 1835	H. dub	0	1	0	2	2	0	2
Linyphiidae	*Diplocephalus latifrons*	(O. P.-Cambridge, 1863)	D. lat	0	0	1	0	5	1	0
Linyphiidae	*Ceratinella brevis*	(Wider, 1834)	C. bre	0	0	0	0	6	1	0
Linyphiidae	*Kaestneria dorsalis*	(Wider, 1834)	K. dor	0	0	0	0	7	0	0
Araneidae	*Nuctenea umbratica*	(Clerck, 1757)	N. umb	0	0	0	0	5	0	1

Table S2 Abundance of all 140 spider species sorted according to their total abundance. Abbrev=Abbreviation of species names. A.g. = *A. glutinosa*, B.p. = *B. pendula*, C.b.= *C. betulus*, P.t. = *P. tremula*, Q.r.= *Q. robur*, P.a. = *P. abies*, P.s.= *P. sylvestris*

Family	Spider species	Author	Abbrev	A.g.	B.p.	C.b.	P.t.	Q.r.	P.a.	P.s.
Thomisidae	*Xysticus audax*	(Schrank, 1803)	X. aud	0	0	0	0	6	0	0
Linyphiidae	*Linyphia triangularis*	(Clerck, 1757)	L. tri	0	0	0	0	6	0	0
Theridiidae	*Dipoena nigroreticulata*	(Simon, 1879)	D. nig	0	0	0	0	6	0	0
Clubionidae	*Clubiona lutescens*	Westring, 1851	C. lut	0	0	0	0	5	0	0
Linyphiidae	*Hylyphantes graminicola*	(Sundevall, 1830)	H. gra	0	0	0	0	5	0	0
Uloboridae	*Hyptiotes paradoxus*	(C.L. Koch, 1834)	H. par	0	0	0	0	1	4	0
Linyphiidae	*Diplocephalus cristatus*	(Blackwall, 1833)	D. cri	0	0	2	0	3	0	0
Clubionidae	*Clubiona subsultans*	Thorell, 1875	C. sub	0	0	0	0	4	1	0
Salticidae	*Evarcha falcata*	(Clerck, 1757)	E. fal	0	0	0	3	0	0	1
Linyphiidae	*Araeoncus humilis*	(Blackwall, 1841)	A. hum	0	2	0	0	2	0	0
Zoridae	*Zora spinimana*	(Sundevall, 1833)	Z. spi	1	0	0	1	2	0	0
Mimetidae	*Ero furcata*	(Villers, 1789)	E. fur	0	0	0	0	3	0	1
Philodromidae	*Philodromus margaritatus*	(Clerck, 1757)	P. mar	0	0	0	0	2	2	0
Theridiidae	*Theridion palmgreni*	Marusik & Tsellarius, 1986	T. pal	0	0	0	0	0	4	0
Theridiidae	*Dipoena melanogaster*	(C.L. Koch, 1837)	D. mel	0	0	0	0	4	0	0
Linyphiidae	*Linyphia hortensis*	Sundevall, 1830	L. hor	0	0	0	0	4	0	0
Linyphiidae	*Oedothorax apicatus*	(Blackwall, 1850)	O. api	0	0	0	0	4	0	0
Linyphiidae	*Porrhomma microphthalmum*	(O. P.-Cambridge, 1871)	P. mic	1	1	0	0	1	0	0
Lycosidae	*Pirata hygrophilus*	Thorell, 1872	P. hyg	2	0	0	0	1	0	0
Clubionidae	*Clubiona caerulescens*	L. Koch, 1867	C. cae	0	0	1	0	2	0	0

Table S2 Abundance of all 140 spider species sorted according to their total abundance. Abbrev=Abbreviation of species names. A.g. = *A. glutinosa*, B.p. = *B. pendula*, C.b.= *C. betulus*, P.t. = *P. tremula*, Q.r.= *Q. robur*, P.a. = *P. abies*, P.s.= *P. sylvestris*

Family	Spider species	Author	Abbrev	A.g.	B.p.	C.b.	P.t.	Q.r.	P.a.	P.s.
Araneidae	*Araneus angulatus*	Clerck, 1757	A. ang	0	0	0	0	2	0	1
Linyphiidae	*Entelecara erythropus*	(Westring, 1851)	E. ery	1	0	0	0	2	0	0
Theridiosomatidae	*Theridiosoma gemmosum*	(L. Koch, 1877)	T. gem	1	0	0	0	2	0	0
Thomisidae	*Ozyptila praticola*	(C.L. Koch, 1837)	O. pra	0	0	0	0	3	0	0
Theridiidae	*Phylloneta impressa*	L. Koch, 1881	T. imp	0	0	0	0	3	0	0
Sparassidae	*Micrommata virescens*	(Clerck, 1757)	M. vir	0	0	0	1	0	0	1
Linyphiidae	*Porrhomma oblitum*	(O. P.-Cambridge, 1871)	P. obl	0	1	0	0	1	0	0
Linyphiidae	*Savignia frontata*	Blackwall, 1833	S. fro	0	1	0	0	0	1	0
Theridiidae	*Euryopis flavomaculata*	(C.L. Koch, 1836)	E. fla	0	0	0	0	0	0	2
Linyphiidae	*Bathyphantes gracilis*	(Blackwall, 1841)	B. gra	1	0	0	0	1	0	0
Araneidae	*Gibbaranea bituberculata*	(Walckenaer, 1802)	G. bit	0	0	0	0	0	2	0
Salticidae	*Heliophanus cupreus*	(Walckenaer, 1802)	H. cup	0	0	0	0	1	0	1
Linyphiidae	*Obscuriphantes obscurus*	(Blackwall, 1841)	O. obs	0	0	0	0	0	2	0
Linyphiidae	*Entelecara acuminata*	(Wider, 1834)	E. acu	0	0	0	0	1	1	0
Linyphiidae	*Gnathonarium dentatum*	(Wider, 1834)	G. den	0	0	0	0	2	0	0
Linyphiidae	*Hypomma bituberculatum*	(Wider, 1834)	H. bit	0	0	0	0	2	0	0
Theridiidae	*Robertus lividus*	(Blackwall, 1836)	R. liv	0	0	0	0	2	0	0
Araneidae	*Gibbaranea omoeda*	(Thorell, 1870)	G. omo	0	0	0	0	2	0	0
Gnaphosidae	*Haplodrassus silvestris*	(Blackwall, 1833)	H. sil	0	0	0	0	2	0	0
Linyphiidae	*Tenuiphantes flavipes*	(Blackwall, 1854)	T. fla	0	0	0	0	2	0	0

Table S2 Abundance of all 140 spider species sorted according to their total abundance. Abbrev=Abbreviation of species names. A.g. = *A. glutinosa*, B.p. = *B. pendula*, C.b.= *C. betulus*, P.t. = *P. tremula*, Q.r.= *Q. robur*, P.a. = *P. abies*, P.s.= *P. sylvestris*

Family	Spider species	Author	Abbrev	A.g.	B.p.	C.b.	P.t.	Q.r.	P.a.	P.s.
Linyphiidae	*Walckenaeria atrotibialis*	(O. P.-Cambridge, 1878)	W. atr	0	0	0	0	2	0	0
Philodromidae	*Philodromus rufus*	Walckenaer, 1826	P. ruf	0	0	0	1	0	0	0
Thomisidae	*Xysticus cristatus*	(Clerck, 1757)	X. cri	0	1	0	0	0	0	0
Theridiidae	*Robertus arundineti*	(O. P.-Cambridge, 1871)	R. aru	0	1	0	0	0	0	0
Theridiidae	*Phylloneta sisyphia*	(Clerck, 1757)	T. sis	0	1	0	0	0	0	0
Araneidae	*Araneus diadematus*	Clerck, 1757	A. dia	1	0	0	0	0	0	0
Theridiidae	*Neottiura bimaculata*	(Linnaeus, 1767)	N. bim	0	0	0	0	0	0	1
Tetragnathidae	*Pachygnatha listeri*	Sundevall, 1830	P. lis	1	0	0	0	0	0	0
Linyphiidae	*Silometopus elegans*	(O. P.-Cambridge, 1872)	S. ele	1	0	0	0	0	0	0
Tetragnathidae	*Tetragnatha pinicola*	L. Koch, 1870	T. pin	1	0	0	0	0	0	0
Philodromidae	*Tibellus oblongus*	(Walckenaer, 1802)	T. obl	0	0	0	0	0	0	1
Gnaphosidae	*Haplodrassus soerenseni*	(Strand, 1900)	H. soe	0	0	1	0	0	0	0
Theridiidae	*Robertus neglectus*	(O. P.-Cambridge, 1871)	R. neg	1	0	0	0	0	0	0
Linyphiidae	*Tenuiphantes alacris*	(Blackwall, 1853)	T. ala	0	0	1	0	0	0	0
Clubionidae	*Clubiona terrestris*	Westring, 1851	C. ter	0	0	0	0	1	0	0
Linyphiidae	*Dismodicus bifrons*	(Blackwall, 1841)	D. bif	0	0	0	0	1	0	0
Linyphiidae	*Gongylidiellum murcidum*	Simon, 1884	G. mur	0	0	0	0	1	0	0
Linyphiidae	*Microlinyphia pusilla*	(Sundevall, 1830)	M. pus	0	0	0	0	1	0	0
Theridiidae	*Steatoda bipunctata*	(Linnaeus, 1758)	S. bip	0	0	0	0	1	0	0
Linyphiidae	*Tiso vagans*	(Blackwall, 1834)	T. vag	0	0	0	0	1	0	0

Table S2 Abundance of all 140 spider species sorted according to their total abundance. Abbrev=Abbreviation of species names. A.g. = *A. glutinosa*, B.p. = *B. pendula*, C.b.= *C. betulus*, P.t. = *P. tremula*, Q.r.= *Q. robur*, P.a. = *P. abies*, P.s.= *P. sylvestris*

Family	Spider species	Author	Abbrev	A.g.	B.p.	C.b.	P.t.	Q.r.	P.a.	P.s.
Linyphiidae	*Walckenaeria alticeps*	(Denis, 1952)	W. alt	0	0	0	0	0	1	0
Linyphiidae	*Oedothorax gibbosus*	(Blackwall, 1841)	O. gib	0	0	0	0	1	0	0
Linyphiidae	*Cnephalocotes obscurus*	(Blackwall, 1834)	C. obs	0	0	0	0	1	0	0
Linyphiidae	*Diplostyla concolor*	(Wider, 1834)	D. con	0	0	0	0	1	0	0
Linyphiidae	*Drapetisca socialis*	(Sundevall, 1833)	D. soc	0	0	0	0	1	0	0
Theridiidae	*Episinus angulatus*	(Blackwall, 1836)	E. ang	0	0	0	0	1	0	0
Salticidae	*Heliophanus flavipes*	(Hahn, 1832)	H. fla	0	0	0	0	1	0	0
Linyphiidae	*Meioneta mollis*	(O. P.-Cambridge, 1871)	M. mol	0	0	0	0	1	0	0
Linyphiidae	*Micrargus subaequalis*	(Westring, 1851)	M. sub	0	0	0	0	1	0	0
Linyphiidae	*Microneta viaria*	(Blackwall, 1841)	M. via	0	0	0	0	1	0	0
Dictynidae	*Nigma flavescens*	(Walckenaer, 1830)	N. fla	0	0	0	0	1	0	0
Linyphiidae	*Oedothorax retusus*	(Westring, 1851)	O. ret	0	0	0	0	1	0	0
Linyphiidae	*Peponocranium orbiculatum*	(O. P.-Cambridge, 1882)	P. orb	0	0	0	0	0	1	0
Thomisidae	*Pistius truncatus*	(Pallas, 1772)	P. tru	0	0	0	0	1	0	0
Salticidae	*Salticus cingulatus*	(Panzer, 1797)	S. cin	0	0	0	0	1	0	0
Philodromidae	*Thanatus sabulosus*	(Menge, 1875)	T. sab	0	0	0	0	1	0	0
Theridiidae	*Theridion melanurum*	Hahn, 1831	T. mel	0	0	0	0	1	0	0
Linyphiidae	*Walckenaeria nudipalpis*	(Westring, 1851)	W. nud	0	0	0	0	1	0	0
Linyphiidae	*Walckenaeria cuspidata*	Blackwall, 1833	W. cus	0	0	0	0	1	0	0
Thomisidae	*Xysticus ferrugineus*	Menge, 1876	X. fer	0	0	0	0	1	0	0
